# Near cut-off wavelength operation of resonant waveguide grating biosensors

**DOI:** 10.1038/s41598-021-92327-4

**Published:** 2021-06-22

**Authors:** Balint Kovacs, Fabio Aldo Kraft, Zsolt Szabo, Yousef Nazirizadeh, Martina Gerken, Robert Horvath

**Affiliations:** 1 Nanobiosensorics Laboratory, ELKH EK MFA, Budapest, Hungary; 2 Division of Medical Image Computing (MIC), German Cancer Research Center (DKFZ), Heidelberg, Germany; 3 Medical Faculty Heidelberg, Heidelberg University, Heidelberg, Germany; 4 Institute of Electrical Engineering and Information Technology, Kiel University, Kiel, Germany; 5 Faculty of Information Technology and Bionics, Pázmány Péter Catholic University, Budapest, Hungary; 6 Byonoy GmbH, Hamburg, Germany

**Keywords:** Biophysics, Applied physics, Biomedical engineering, Optical sensors

## Abstract

Numerical simulations and analytical calculations are performed to support the design of grating-coupled planar optical waveguides for biological sensing. Near cut-off and far from cut-off modes are investigated, and their characteristics and suitability for sensing are compared. The numerical simulations reveal the high sensitivity of the guided mode intensity near the cut-off wavelength for any refractive index change along the waveguide. Consequently, it is sufficient to monitor the intensity change of the near cut-off sensing mode, which leads to a simpler sensor design compared to those setups where the resonant wavelength shift of the guided mode is monitored with high precision. The operating wavelength and the sensitivity of the proposed device can be tuned by varying the geometrical parameters of the corrugated waveguide. These results may lead to the development of highly sensitive integrated sensors, which have a simple design and therefore are cost-effective for a wide range of applications. These numerical findings are supported with experimental results, where the cut-off sensing mode was identified.

## Introduction

Optical biosensors based on the evanescent field of surface plasmons^1–4^, optical waveguide interferometers^2,3^, photonic crystals^2^ or resonant waveguide gratings (RWG)^3,5^ are widely utilized to directly analyze biochemical and cellular interactions without the need of any biomolecular labels. Recent developments increased the sensitivity of these sensors and made them especially popular not only in drug discovery, but also for biological measurements involving even single cells^1–5^.

The most common evanescent wave biosensors are the surface plasmon resonance (SPR) devices, which employ the evanescent field of the light-excited surface plasmon polaritons on the boundary of metal-dielectric interfaces^1,6^. Usually the sensitivity of these devices is limited to the nanomolar range, which is appropriate for diverse biochemical applications^7–9^. However, further research is required to miniaturize these sensors for portable clinical applications^10^.

Optical waveguide interferometers utilize the phase difference between the two arms of the sensor, which is caused by the presence of the sample in the evanescent field of the measuring section. These biosensors can offer a broad sensing range, but the modes at different wavelengths have different sensitivity due to their different propagation speed and penetration depth. This behavior can decrease the information content of the signal. Therefore, this technique is more appropriate for biomolecular detection^11,12^.

Photonic crystals^13,14^ are periodic structures usually made of two dielectrics with characteristic dimensions comparable to the working wavelength region. The multiple reflections due to the periodic refractive index contrast can completely cancel the wave propagation, and can lead to photonic bandgaps^15^. Photonic crystal waveguides and cavities have been applied for cell adhesion research^16,17^ and also in cellular-level drug discovery^18^.

The steady increase of cell-based assays for early drug discovery with high throughput and sensitivity in the picomolar range made the RWG one of the most used optical biosensor in our days^5,19^. Due to its simple structure, it can be easily combined with other biosensor devices, which enables even single-cell adhesion measurements^20,21^. Like other biosensors, it measures the refractive index changes near the sensor surface by following the changes of the resonant wavelength, but it has the advantage that for TM_0_ mode the penetration depth is strongly confined to the cell-substrate contact zone, which is crucial for cell adhesion measurements^22,23^. Measurements with the lowest limit of detection have been achieved using interferometric readout^24,25^. However, the interferometric method as well as the common measurement method with wavelength analysis significantly increase the cost of this biosensor and makes its miniaturization difficult. This limits its applicability in the everyday clinical practice. To overcome this limitation, an intensity-based measuring method has been developed by operating the sensor near the so-called cut-off frequency. In this case, the change of the assay (cover) refractive index has a significant impact on the intensity of the guided mode^26–28^. The guided mode disappears when the effective refractive index *N* reaches the cut-off point. For a normal-symmetry waveguide the relation ${n}_{F}>{n}_{S}>{n}_{C}$ holds for the relative magnitude of the high-index waveguide-film refractive index ${n}_{F}$, the substrate refractive index ${n}_{S}$ and the cover refractive index ${n}_{C}$^29^. For a reverse-symmetry waveguide we have ${n}_{F}>{n}_{C}>{n}_{S}$ and *N* is limited by the refractive index of the waveguide film ${n}_{F}$ and of the cover media ${n}_{C}$. The guided mode disappears when *N* reaches one of the boundaries of this range^30^. The disappearance of the mode could be followed by a simple intensity measurement^26,28^.

There are already miniaturized readout systems available, which can convert a wavelength shift into an intensity change^31,32^. Integration of the RWG, which operates near the cut-off point with such readout systems can provide commercially available clinical sensors within foreseeable time because their sensitive and flexible measurement method for a wide sensing range^33,34^. Despite of the advantages of the RWG sensors, which could be operated near the cut-off point, their operational conditions have never been in-depth analyzed.

In this work, we present the electromagnetic design of RWG sensors, which operate near the substrate cut-off point. Simulations are performed with commercial electromagnetic solvers and the guided-mode resonances are also calculated analytically with the equations of the 3-layer model. The changes of the position and of the intensity for ‘far from cut-off’ and ‘near cut-off’ reflection resonant peaks as a function of the waveguide thickness and of the refractive index of the cover media are investigated. The electromagnetic field distributions of the sensor are visualized near the resonances, the penetration depth of the evanescent field is determined and the sensitivity is also calculated. The simulations demonstrate that by changing only one geometrical parameter, the grating depth of the RWG sensor can be conveniently tailored to the needs of different applications. We also perform experimental reflection measurements with grating waveguides to validate the key numerical findings.

## Methods

### The modeled waveguide structure

**Figure 1:**
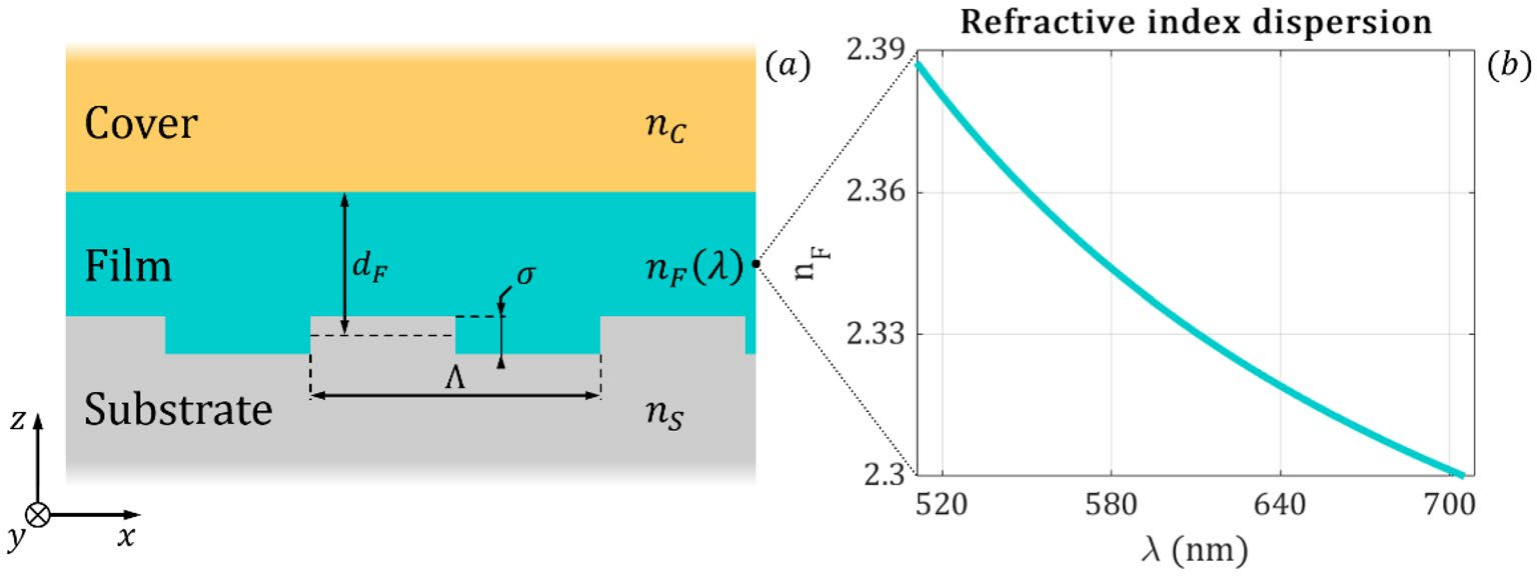
**(a)** The cross-section of the modeled corrugated waveguide. The electromagnetic simulations apply Floquet boundary conditions along the x- and y-axis and open boundaries with Floquet ports in the vertical z direction. **(b)** The frequency dependence of the Nb_2_O_5_ film refractive index.

The cross-section of the modeled corrugated planar optical waveguide is presented in Fig. 1a. The substrate has a refractive index of ${n}_{S}=1.54$, and it is covered by a Nb_2_O_5_ (niobium pentoxide) film with refractive index ${n}_{F}$ and thickness ${d}_{F}$. The calculations take into account the wavelength dependence of the Nb_2_O_5_ film refractive index as it shown in Fig. 1b^35,36^. The waveguide film is considered to be covered by an aqueous medium with refractive index ${n}_{C}$. The period of the square profiled grating is *Λ* = 480 nm, the depth is *σ* = 50 nm and it is located at the substrate–film boundary.

### Simulation environment and implementation

The electromagnetic simulations are performed by the Frequency Domain Solver of the commercial simulation environment CST Studio Suit^37^. This solver is based on the finite element method with tetrahedral mesh, and it is suitable for the simulations of electrically small periodic resonant structures. It can provide the electromagnetic near- and far-fields as well as the S-parameters (transmission and reflection data). The finite element solver is equipped with Floquet boundary conditions, which is called Unit Cell Boundary condition in the settings, therefore it is enough to simulate one period of the corrugated waveguide for a plane wave excitation with any wave vector confined in the x–z plane. To keep the mesh size as small as possible, in the y direction only a thin portion of the geometry as compared to the wavelength is considered. Along the horizontal boundaries (x and y directions) the Floquet boundary conditions, while in the vertical *z* direction open boundaries implemented with the Floquet ports are set. The TE modes or the TM modes can be set by selecting the proper excitation of the Floquet ports.

Plane wave excitation of the structure with transverse magnetic (TM) and transverse electric (TE) light—where the magnetic/electric field vector is parallel to the interfaces of the media, respectively—is introduced by the Floquet port with an incidence angle of 26°. For sensing purposes, it is beneficial to use the TM mode over the TE mode due to its larger sensitivity for any refractive index change close to the sensor surface^38^. The Epic® RWG biosensor, which is a leading product among RWG based biosensors^19,39^ also measures the resonant wavelength of the zeroth order TM mode.

During the numerical simulations, the reflection spectra (S_1,1_ parameter) and the electromagnetic field distribution are investigated. The reflection is calculated at four different waveguide film thicknesses d_F_ = 175 nm, 180 nm, 185 nm, 190 nm with varying ${n}_{C}$ from 1.27 to 1.45 with steps 0.01 using the parameter sweep function. Moreover, simulations are run for *σ* = 20nm, 50 nm, 80 nm grating depths. Field monitors are set at the wavelength of the reflection peaks to examine the distribution of the electromagnetic field near the surface of the sensor. A detailed discussion of the relative wavelength position of the reflection peak with respect to the mode wavelength is given in Ref.^40^. In case of the TM excitation the magnetic field is continuous over the boundaries of different media therefore it can provide an insight of the RWG sensor functionality. However, the biomolecules and the cells are nonmagnetic structures therefore they interact with the electric field. Consequently, the electric field distributions are visualized when the penetration depth into the cover media is investigated.

## Results and discussion

### Investigation of the RWG modes suitable for sensing

**Figure 2:**
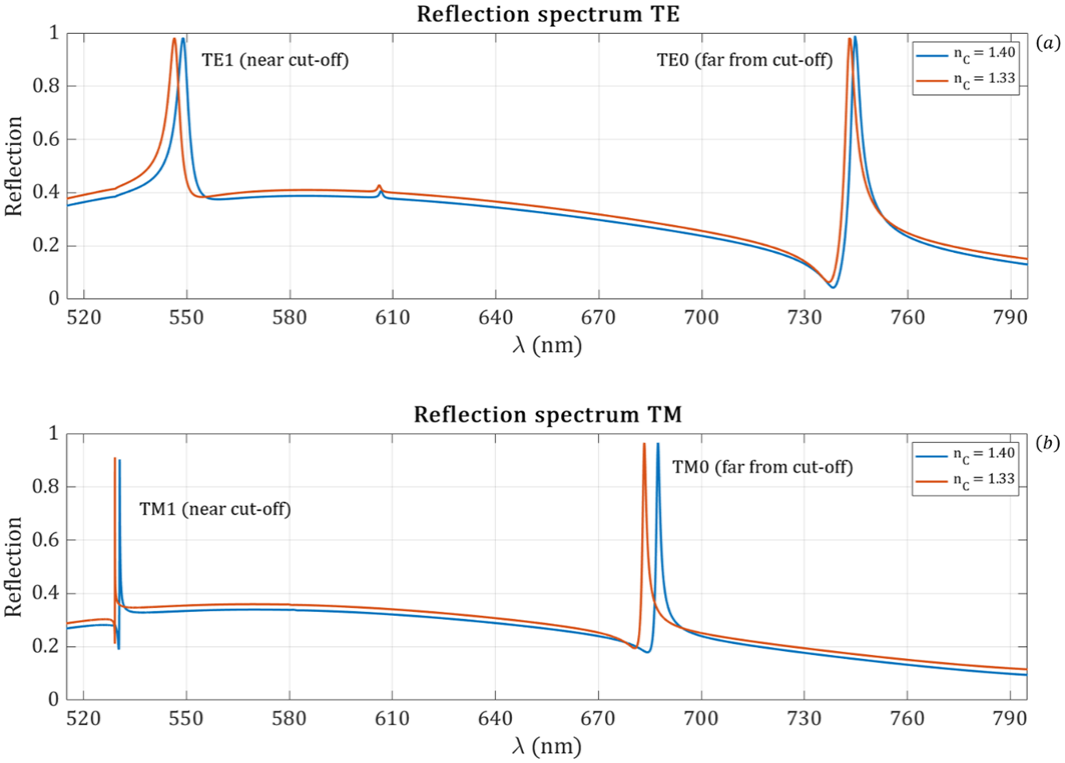
Simulated reflection spectra of the sensor for TE and TM excitation with d_*F*_ = 185 nm and for two different cover refractive indices with an incidence angle of 26°. (**a**) The TE reflection spectrum of the sensor. The right peak corresponds to the ${TE}_{0}$ mode, while the left peak corresponds to the ${TE}_{1}$ mode. (**b**) The TM reflection spectrum of the sensor. The right peak corresponds to the ${TM}_{0}$ mode, while the left peak corresponds to the ${TM}_{1}$ mode. It can be seen that the resonant peaks are shifted due to the cover refractive index change but not only the wavelength shift of the ${TM}_{1}$ peak is smaller compare to the other resonant peaks but it became also narrower.

First, the reflection spectra are simulated by employing the settings discussed in “Simulation environment and implementation” for both TE and TM excitation with d_*F*_ = 185 nm and for two different cover refractive indices. The obtained reflection spectra are shown in Fig. 2. As it can be observed in the simulated wavelength range, the reflection curves have two dominant peaks each. In the TE spectrum a small peak is visible at ~ 600 nm. It can also be seen that the resonant peaks are shifted due to the cover refractive index change but not only the wavelength shift of the ${TM}_{1}$ peak is smaller compare to the other resonant peaks but it became also narrower.

For further analysis, the reflection spectrum is calculated for various waveguide film thicknesses in the range of 175–190 nm and the position of the resonant peaks are compared with the peak positions directly calculated from the equation of the 3-layer model^38^

1 $${d}_{F}\frac{2\pi }{\lambda }\sqrt{{n}_{F}^{2}-{N}^{2}}-\mathit{arctan}\left[{\left(\frac{{n}_{F}}{{n}_{C}}\right)}^{2\rho }\frac{\sqrt{{N}^{2}-{n}_{C}^{2}}}{\sqrt{{n}_{F}^{2}-{N}^{2}}}\right]-arctan\left[{\left(\frac{{n}_{F}}{{n}_{S}}\right)}^{2\rho }\frac{\sqrt{{N}^{2}-{n}_{S}^{2}}}{\sqrt{{n}_{F}^{2}-{N}^{2}}}\right]=\pi m$$

where ρ = 0 for TE polarization, ρ = 1 for TM polarization and an integer m is the order of excited mode. In order to calculate the wavelengths of the guided modes for a given illumination angle $\alpha$, the effective refractive index of Eq. (1) is calculated from the grating equation:

2 $$N=\mathit{sin}\left(\alpha \right)+l\cdot \frac{\lambda }{\Lambda }$$

where $\alpha$ is the incidence angle of the excitation, $l$ is the coupled diffraction order, $\lambda$ is the excitation wavelength and $\Lambda$ is the grating period. From this analysis it follows that the right peaks correspond to the 0th order mode in the first diffraction order (*m* = 0, *l* = 1), while the left peaks correspond to the 1st order mode (*m* = 1, *l* = 1). The small TE peak at ~ 600 nm is the second diffraction order of the 0th order mode (*m* = 0, *l* = 2). There is a resonant wavelength shift of the peaks caused by the changed cover refractive index. However, the wavelength shift of the $\mathrm{T}{\mathrm{M}}_{1}$ peak is much smaller compared to the other resonant peaks and its form is also changed for the refractive index change. In the followings, we are investigating this behavior of the TM_1_ resonant peak focusing on the TM excitation of the sensor structure. In all the following calculations the first diffraction order is considered ($l=1$).

**Figure 3:**
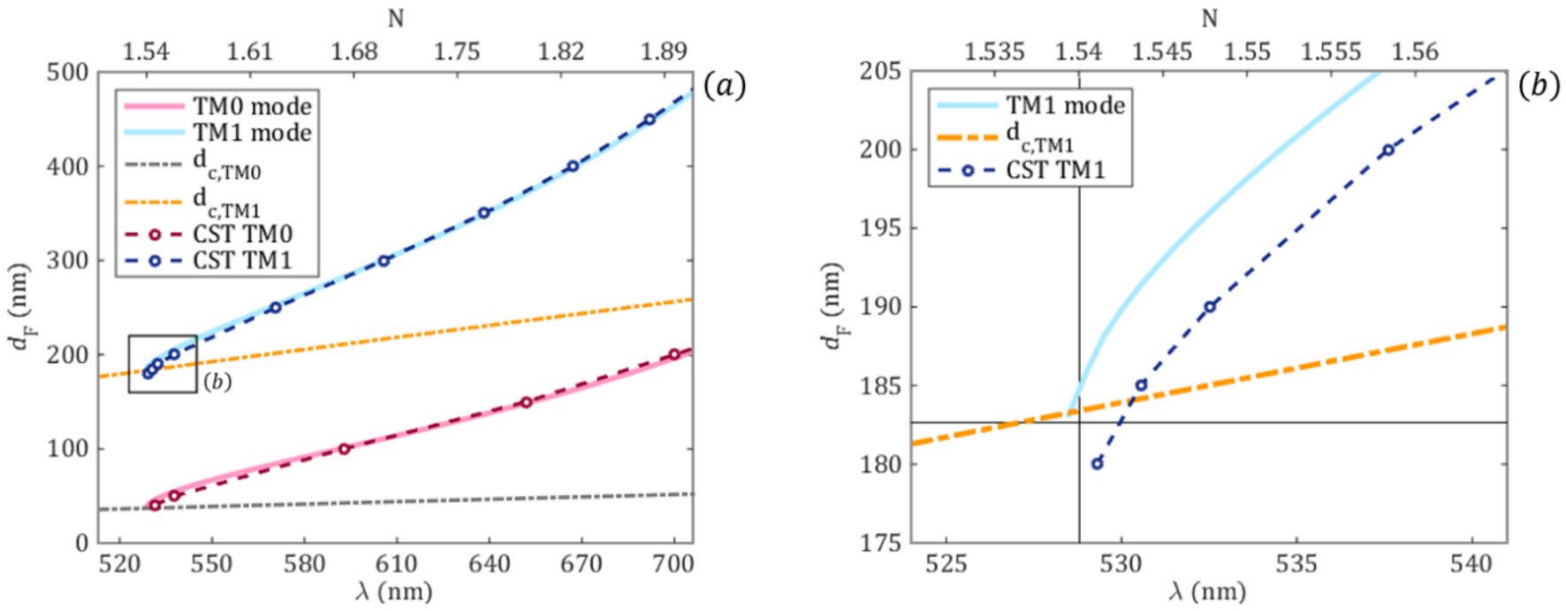
(**a**) TM_0_ and TM_1_ mode curves calculated with the 3-layer model (continuous lines) and the numerical electromagnetic simulations (open circles) with an incidence angle of 26°. The graph shows the waveguide thicknesses as function of the corresponding illumination wavelength and effective refractive index when the cover refractive index is ${\mathrm{n}}_{\mathrm{C}}=1.4$. The dashed lines are the thicknesses corresponding to the cut-off wavelengths. (**b**) Magnification of the TM_1_ curve near the cut-off point. The black vertical line is located at $\mathrm{N}={\mathrm{n}}_{\mathrm{S}}=1.54$, which is the cut-off point.

The cut-off waveguide film thickness is calculated for the two TM modes using the equation:

3 $${d}_{c}=\frac{1}{{k}_{z}\sqrt{{n}_{F}^{2}-{n}_{max}^{2}}}\cdot \left(\mathrm{arctan}\left[{\left(\frac{{n}_{F}}{{n}_{min}}\right)}^{2\rho }\cdot \sqrt{\frac{{n}_{max}^{2}-{n}_{min}^{2}}{{n}_{F}^{2}-{n}_{max}^{2}}}\right]+\pi m\right)$$

where ${n}_{min}=min\left\{{n}_{S},{n}_{C}\right\}$, ${n}_{max}=max\left\{{n}_{S},{n}_{C}\right\}$^30^_._ For the modeled structure we consider ${n}_{max}={n}_{S}$ and ${n}_{min}={n}_{C}$. It is important to note that the specified mode cannot exist in the structure if ${d}_{F}<{d}_{c}$. The results of the analytical calculations and a comparison with numerical simulations are summarized in Fig. 3. The numerically obtained left and right peaks of Fig. 2 correspond to the TM_1_ and TM_0_ mode, respectively. More importantly, by decreasing the thickness of the waveguide film, the TM_1_ resonance shifts closer and closer to the cut-off point and this mode cannot exist when the waveguide thickness is less than 183 nm. This thickness corresponds to the cut-off of the TM_1_ mode, where it turns from a propagating to evanescent wave. However, the TM_0_ mode stably exists even for decreased film thicknesses. Therefore, we refer to the $\mathrm{T}{\mathrm{M}}_{1}$ mode as near cut-off and to the $\mathrm{T}{\mathrm{M}}_{0}$ mode as far-from-cut-off resonances. The cut-off points are d_F_ = 183 nm in case of TM_1_ and d_F_ = 40 nm for TM_0_.

**Figure 4:**
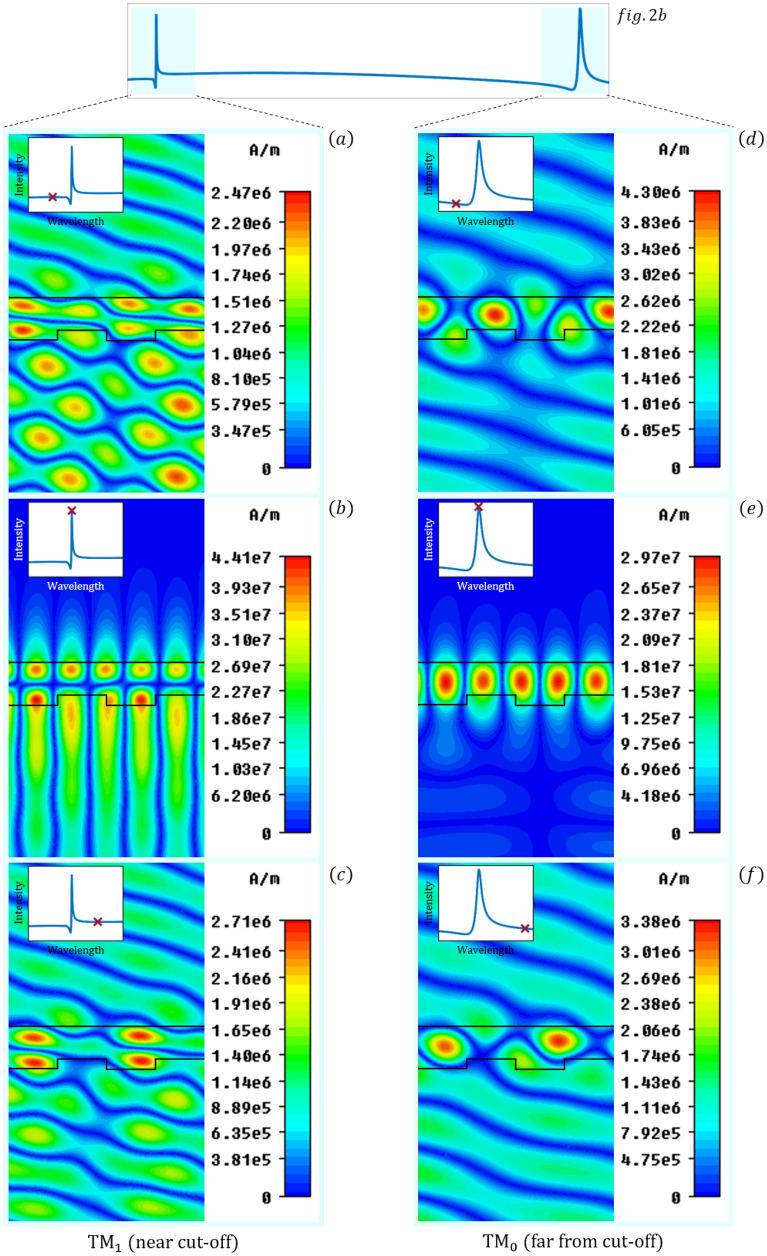
The magnetic field distributions before, at and after the resonant peak of the TM_0_ and TM_1_ resonances. The TM wave incident from the substrate side with an incidence angle of 26° produces field enhancement in the waveguide and an evanescent field at the sensor surface at the resonant wavelengths upon full reflection. It transmits through the grating structure at the off-resonance wavelengths. The stronger localization of the mode power inside the waveguide film for the TM_0_ far-from cut-off mode is observed. The magnetic field distributions are calculated by CST Studio Suit^37^.

The magnetic field distribution of the RWG sensor corresponding to the wavelengths at the maximum amplitudes of the reflection peaks are plotted in Fig. 4. For comparison off-resonance magnetic field distributions are plotted as well. The operating wavelengths are *λ*_*TM0*_ = 678, 688, 698 nm and *λ*_*TM1*_ = 521, 531, 541 nm. At resonance, the exiting TM wave couples to the waveguide mode resulting in field enhancement in the waveguide as well as an evanescent field at the sensor surface. Constructive interference in reflection and destructive interference in transmission is present. The exciting wave passes the grating structure at the off-resonance wavelengths. The second row of the field distributions reveals the difference between the two guided modes. The TM_0_ mode is much stronger localized inside the waveguide film compared to the TM_1_ mode, which is a consequence of the mode profile caused by the geometry of the waveguide^28–30^. To better visualize the effect, the electromagnetic field animations at the resonances can be found in the Supplementary materials.

**Figure 5:**
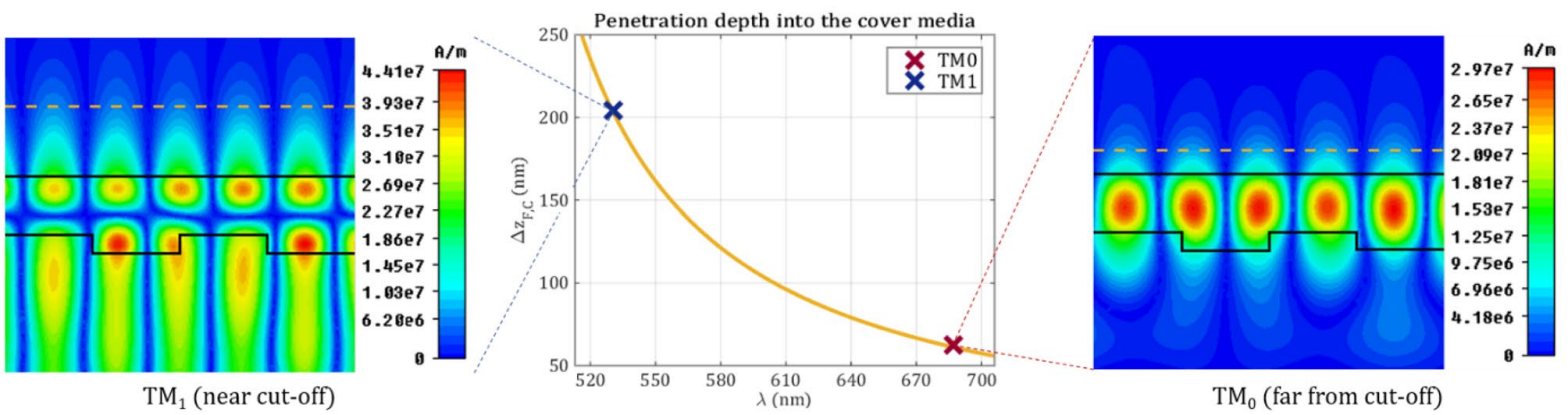
The penetration depth into the cover medium as a function of wavelength for d_F_ = 185 nm and cover refractive index ${\mathrm{n}}_{\mathrm{C}}=1.4$ and with an incidence angle of 26°. The penetration depth of the TM_0_ mode is 61 nm and of the TM_1_ mode is 203 nm as indicated with the cross marks. These values are also visualized on the corresponding magnetic field distributions with orange dashed lines. The magnetic field distributions are calculated by CST Studio Suit^37^.

An essential parameter of surface-sensitive sensor applications is the penetration depth into the aqueous cover media. The analyte biomolecules and living cells on top of the sensor are monitored by the evanescent field of the excited mode. Consequently, for larger penetration depths larger objects or a larger portion of the objects is sensed by the evanescent wave^41^. Therefore, it is important to tailor the penetration depth to needs of the actual application. The penetration depth into the cover medium can be calculated from the following equation^30^

4 $$\Delta {z}_{F,C}=\frac{1-\varrho }{{k}_{z}\sqrt{{N}^{2}-{n}_{C}^{2}}}+\frac{\varrho {\left[{\left(\frac{N}{{n}_{F}}\right)}^{2}+{\left(\frac{N}{{n}_{C}}\right)}^{2}-1\right]}^{-1}}{{k}_{z}\sqrt{{N}^{2}-{n}_{C}^{2}}}$$

where ${k}_{z}$ denotes the component of the wave vector in the z-direction (the direction of mode propagation). In Fig. 5 the penetration depth and the magnetic field distribution of the TM_0_ and TM_1_ modes are plotted. As it can be observed a larger penetration depth is obtained for the TM_1_ mode (203 nm vs. 61 nm). Therefore, the TM_1_ mode is more suitable to sense larger biological objects and can monitor the changes of the refractive index further away from the waveguide surface.

### Sensitivity analysis of the sensor

After investigating the main characteristics of the two dominant resonance peaks, in this section the sensitivity of the sensor is evaluated in function of the refractive index of the cover. The effective refractive index *N* can be decreased to ${n}_{s}$ by decreasing ${n}_{c}$, therefore the normal symmetry waveguide can operate close to the cut-off point^26^.

**Figure 6:**
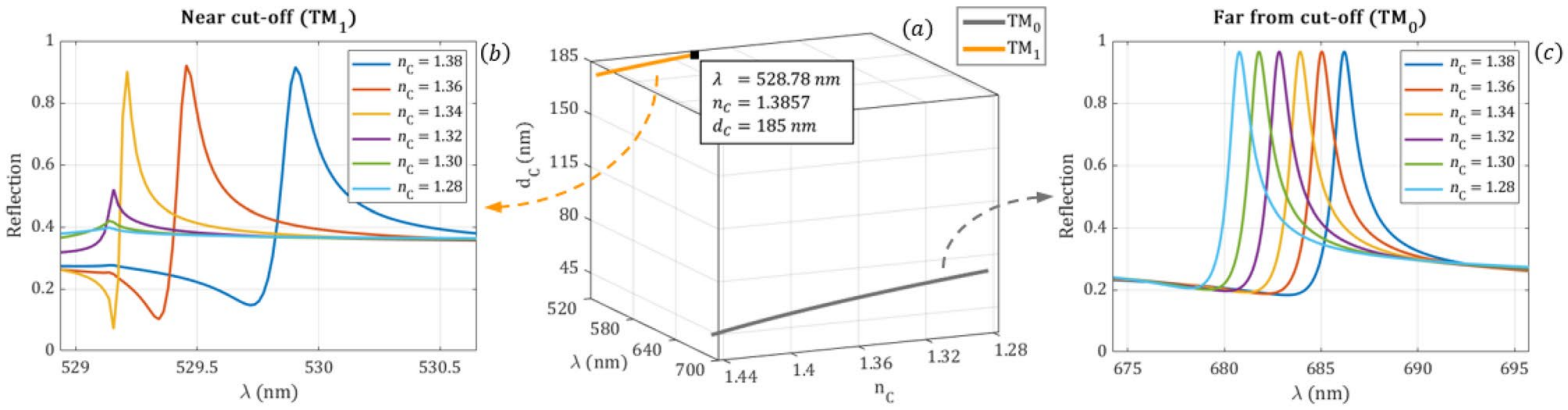
(**a**) The ${\mathrm{d}}_{\mathrm{c}}$ cut-off curves and the resonance wavelengths for the TM_0_ and TM_1_ modes with an incidence angle of 26° for decreasing ${\mathrm{n}}_{\mathrm{C}}$ from the analytical calculations. (**b**,**c**) The reflection spectra of the TM_0_ and TM_1_ modes for several values of the cover refractive indices from the numerical simulations. Changing the cover refractive index not only shifts the location of the resonant peaks, but approaching the cut-off point at λ ≈ 529.1 nm the intensity of the TM_1_ mode decreases sharply while the wavelength shift became less significant. It can also be seen that the TM_1_ mode disappears continuously around the cut-off point in a certain range of ${\mathrm{n}}_{\mathrm{C}}$.

The cut-off waveguide thickness and the resonance wavelength of the modes are calculated using the analytical Eqs. (1–3) by changing ${n}_{C}=1.28-1.45$ and keeping d_*F*_ = 185 nm. The wavelength shift for the two TM modes is shown in Fig. 6a. As it can be observed the cut-off point of the TM_1_ mode is at ${\mathrm{n}}_{\mathrm{C}}=1.3857$. The guided wavelength at the cut-off point of the TM_1_ mode is *λ* = 528.78 nm. The reflection spectrum of the modes calculated with the full/wave electromagnetic solver for different ${n}_{c}$ values is plotted in Fig. 6b,c. As it can be observed the wavelength shift is continuous, and the intensity of the peeks are constant in case of the TM_0_ mode. However, by approaching the cut-off point at λ ≈ 529.1 nm the wavelength shift of the TM_1_ mode decreases and this peak disappears.

**Figure 7:**
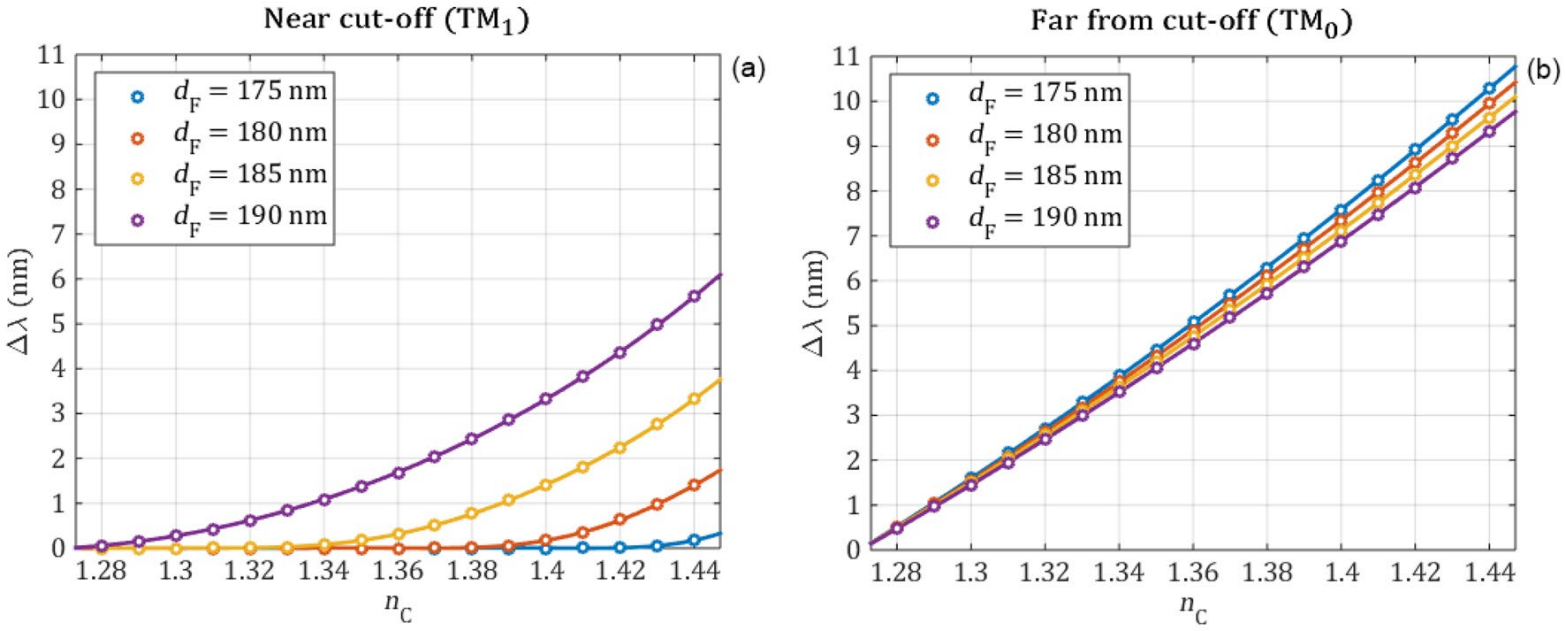
The guided-mode resonance wavelength shifts obtained from full wave electromagnetic simulations (circle markers) are fitted with polynomials (continuous curves) for the near cut-off (**a**) and far-from cut-off (**b**) modes in function of the refractive index of the cover medium for different waveguide film thicknesses. The wavelength shifts are relative to the resonant peak at ${\mathrm{n}}_{\mathrm{C}}=1.27$. The wavelength shift is linear in case of the TM_0_ mode. However, the wavelength shift of the TM_1_ peak becomes less significant approaching the cut-off point.

**Figure 8:**
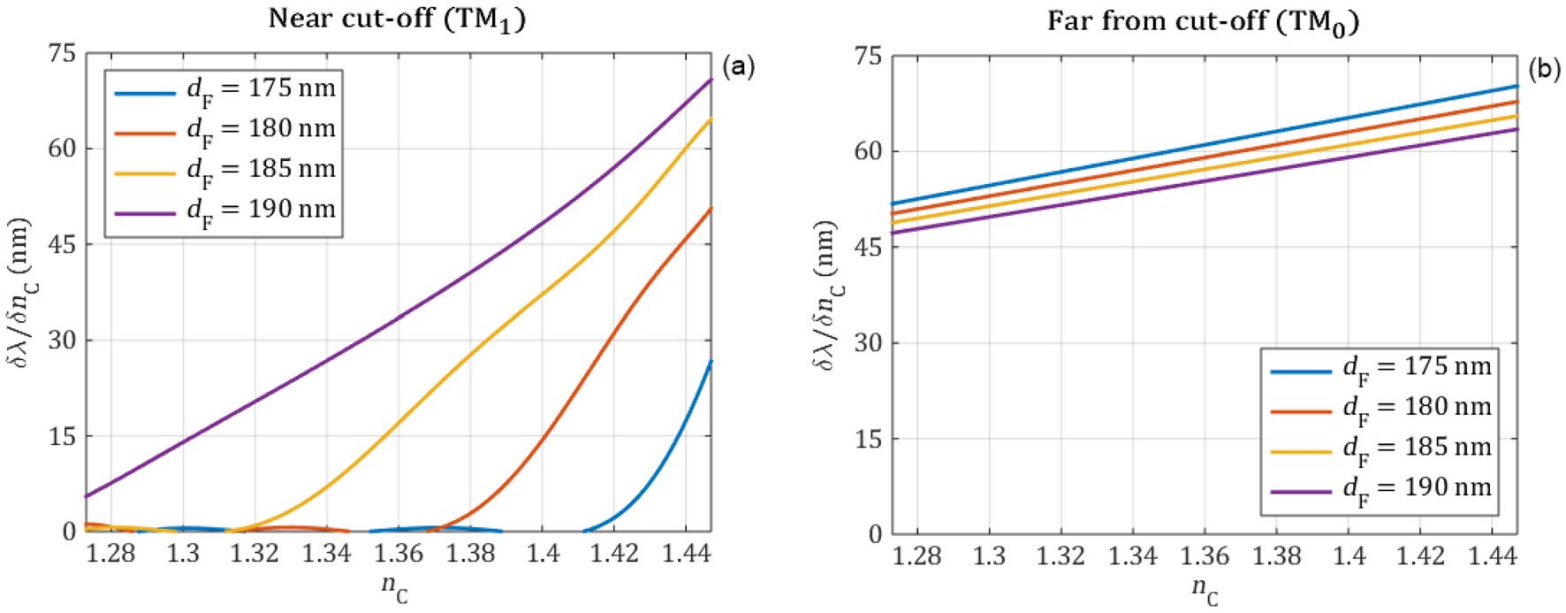
The sensitivity of the wavelength shift as a function of the refractive index of the cover medium for the near cut-off (**a**) and far from cut-off (**b**) resonant peaks at four different waveguide film thicknesses. The curves were created with derivation of a polynomial fitted curves of Fig. 6. It is clearly seen that while the wavelength sensitivity is almost constant in case of TM_0_ (far from cut-off) peak, the sensitivity in case of TM_1_ (near cut-off peak) decreases to zero approaching the cut-off point.

The positions of the resonant peaks are calculated for the waveguide film thicknesses d_F_ = 175 nm, 180 nm, 185 nm, 190 nm by varying the refractive index of the cover medium from 1.27 to 1.45 in steps of 0.01. The wavelength shifts are calculated as the difference to the guided wavelength at ${n}_{C}=1.27$. Polynomial fittings—order of 8 for the near cut-off and 2 for the far from cut-off—are also performed for a better visualization and to determine how the sensitivity of peak position depends on the cover refractive index as it is plotted in Fig. 7. The numerically obtained wavelength shifts are shown with circle markers while the continuous lines are the fitted polynomial curves. The first derivatives of the fitted polynomial curves represent the RI sensitivity and are shown in Fig. 8. It can be observed that the sensitivity is almost constant in case of TM_0_ the mode, which is far from the cut-off, while the sensitivity of the TM_1_ mode decreases to zero as it reaches the cut-off point.

The numerical calculations reveal that the wavelength position of the peaks is highly influenced by the changes of the cover refractive index when the resonant peaks are far from the cut-off point. However, in case of near to the cut-off operation, the wavelength change becomes less significant and it reaches zero at the cut-off point. By modifying the waveguide thickness the operation of the sensor can be shifted. The simulations indicate that due to its linear behavior it is beneficial to use the far-from cut-off resonance for wavelength shift measurements.

**Figure 9:**
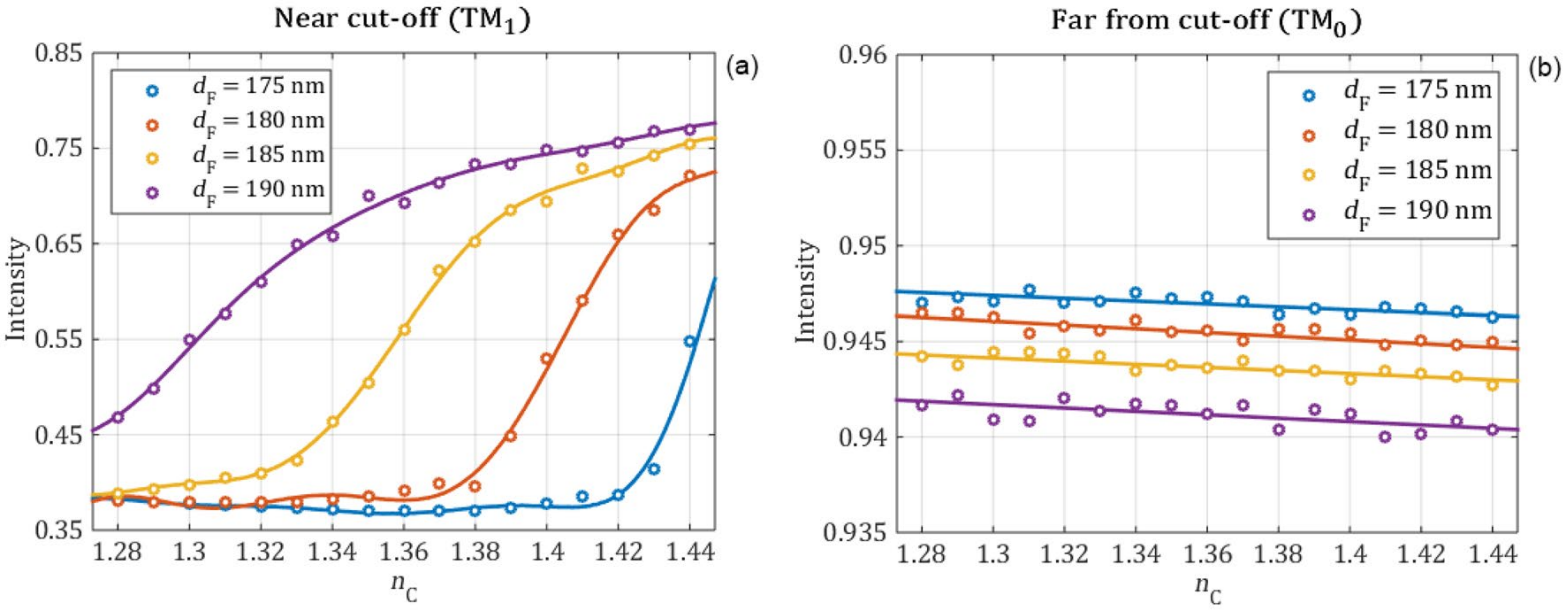
The intensity of the reflection peaks obtained from full wave electromagnetic simulations (circle markers) are fitted with polynomials (continuous curves) for the near cut-off (**a**) and far-from cut-off (**b**) modes in function of the refractive index of the cover media for different waveguide film thicknesses. Far from cut-off the cover refractive index negligibly influences the intensity, while near cut-off the intensity of the mode is highly influenced by the cover refractive index.

**Figure 10:**
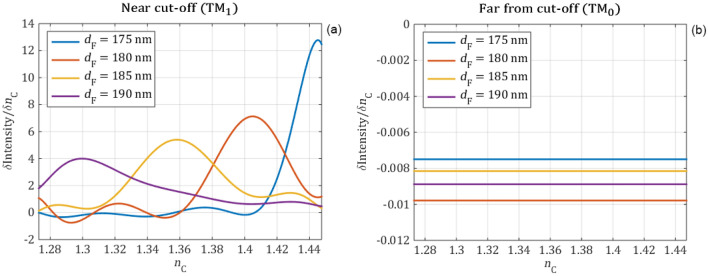
The sensitivity of the intensity difference for the near cut-off (a) and far from cut-off (b) modes as a function of the refractive index of the cover layer for different waveguide film thicknesses.

In the following the intensity change of the reflection peaks is investigated. The intensity values are calculated by integrating a 0.25 nm wide range of the reflection peaks with an excitation amplitude of 1. This range is suitable because it is negligibly influenced by the reflection baseline and it can be easily implemented by a bandpass optical element in a measurement setup. In Fig. 9 the integrated peak intensity values are plotted in the function of the cover refractive index for different waveguide film thicknesses. The numerically obtained peak intensity values are shown with circle markers and the polynomial fittings—order of 9 for the near cut-off and 1 for the far from cut-off—with continuous curves. The first derivatives of the fitted polynomial curves, which represent the sensitivity of the intensity are shown in Fig. 10.

The numerical calculations revealed that far from the cut-off the refractive index changes of the cover medium has almost no effect on the intensity of the reflection peak. However, when the peak is near the cut-off the intensity is highly influenced by the changes of the cover refractive index. By decreasing the refractive index of the cover medium, the intensity of the reflection peak also decreases near the cut-off and it reaches the base reflection intensity at the cut-off point at certain ${\mathrm{n}}_{\mathrm{c}}$ values. Between these two points, the intensity change has a remarkably high sensitivity. The maximal sensitivity and the sensing range can be further tuned by changing the thickness of the waveguide film. Based on these results, we recommend using the TM_1_ peak near cut-off in setups, which measure the intensity.

**Figure 11:**
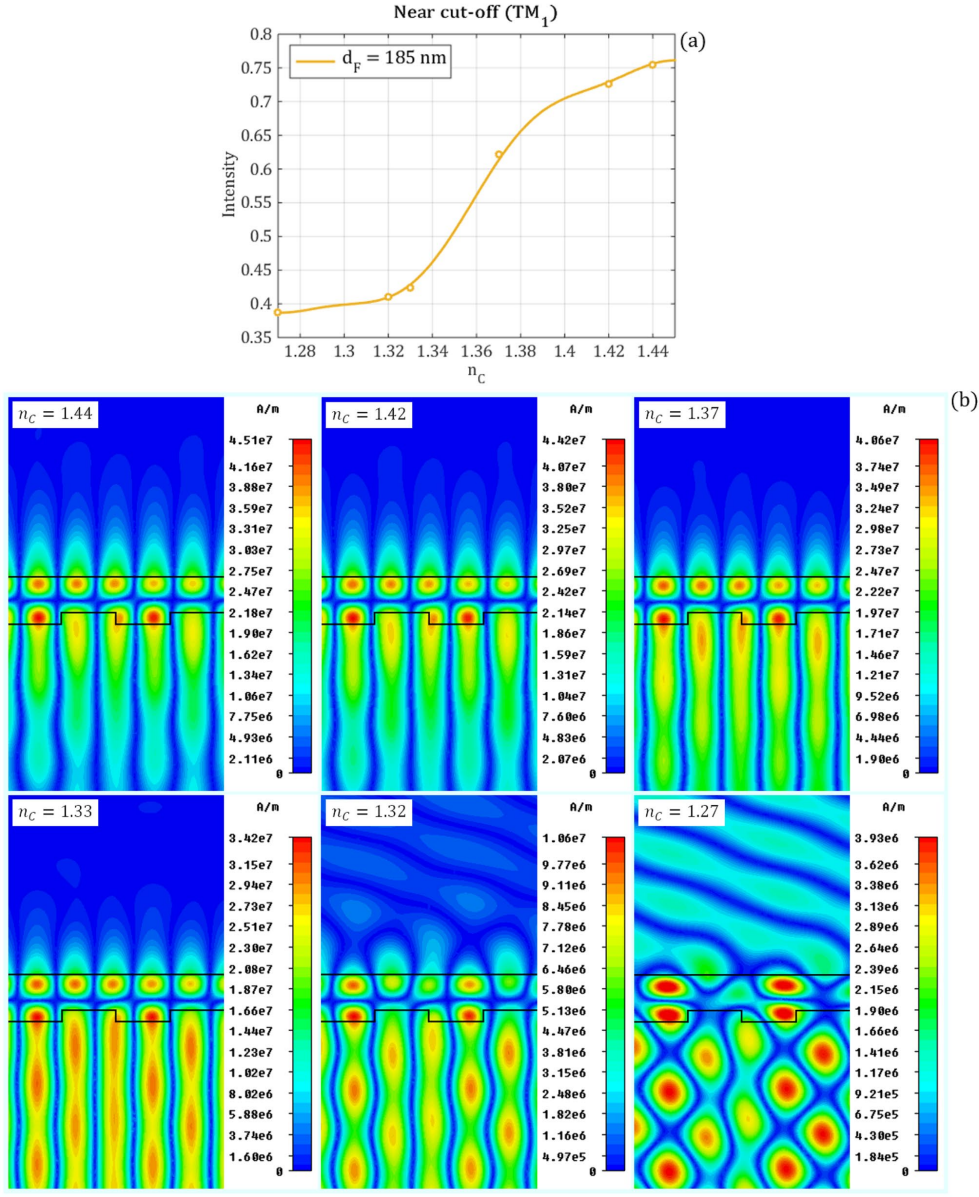
(**a**) The intensity of the near cut-off peak as a function of the refractive index of the cover layer for d_F_ = 185 nm waveguide film thickness. The magnetic field distributions corresponding to the refractive index values marked with circles are shown in (**b**). Decreasing the cover refractive index, the localization of the magnetic field decreases, and after the cut-off point, which is around ${\mathrm{n}}_{\mathrm{C}}=1.33-1.32$ the wave propagates through the waveguide. The magnetic field distributions are calculated by CST Studio Suit^37^.

The waveguide mode does not exists for wavelengths larger than the cut-off point, therefore the resonant peak also disappears at the cut-off point, which results in a large penetration depth into the substrate medium^28–30^. As the mode penetrates further into the substrate the overlap with the corrugation decreases. Thus, the scattering efficiency decreases and an increase in the quality factor is expected. This is observed in Fig. 6b. Numerical simulations near the cut-off resonance are performed for d_F_ = 185 nm while the cover refractive index is decreased to illustrate the behavior of the magnetic field through the cut-off point. The simulated magnetic field distributions for six different ${n}_{C}$ values, which correspond to the intensity curves of Fig. 9a are shown in Fig. 11. The cover refractive index values are selected in such a way to clearly illustrate the characteristics of the field distributions before, at and after the cut-off point. The guided mode disappears around ${n}_{C}=1.33-1.32$ and the localization of the magnetic field decreases as the wave is not confined anymore in the waveguide.

**Figure 12:**
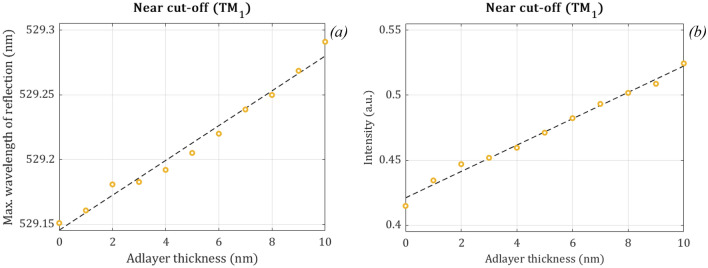
The maximum wavelengths (**a**) and the intensity (**b**) of the reflection peaks for the near cut-off mode in function of the additional biological layer thickness. We also fitted a line on the simulated data (black dashed lines). We used n_C_ = 1.33 as a standard cover refractive index of the puffer solution for biological measurements and d_F_ = 185 nm as before for the main experiments with incident angle of 26°. We changed the thickness (d_A_) of an additional biological layer from 0 to 10 nm on the surface of the sensor with refractive index n_A_ = 1.45, which refractive index value is common for biological samples.

The bulk refractive index sensitivity is an important parameter in refractometry and in living cell based applications. Next, we demonstrate that the near cut-off mode can be also potentially employed to monitor the presence of thin biological layers on the surface of the waveguide. For this purpose, we simulated the same structure with a standard puffer solution for biological measurements with cover refractive index ${n}_{C}=1.33$ and we changed the thickness (${d}_{A}$) of an additional biological layer on the surface of the sensor with refractive index ${n}_{A}=1.45$, which value is general for biological samples. The resulted wavelengths and intensities can be seen in Fig. 12.

We have applied linear regression on the simulated values of maximum wavelengths and intensities of reflection (see Fig. 12 black dashed line) to determine the surface sensitivity of the sensor:

5 $$\lambda ~\left( {{\text{nm}}} \right) = 0.0134 \cdot {\text{d}}_{{\text{A}}} + ~529.1455\;{\text{~nm}}$$

6 $${\text{Intensity}}~\;\left( {{\text{a}}.{\text{u}}.} \right) = 0.0101 \left(1/nm \right) \cdot {\text{d}}_{A} + ~0.4211$$

where the maximum wavelength of the reflection $\mathrm{\lambda }$ and the thickness ${\mathrm{d}}_{A}$ are measured in nm, while the intensity is normalized to the intensity of the incident light. Figure 12 confirms the linearity of the sensor response, an important feature in kinetic measurements and further evaluation^42^.

As we have already shown in this section that the tuning of ${d}_{F}$ has a huge effect not only on the location of the cut-off point but also on the sensitivity of the peak intensity. It is already shown that the shallower the grating, the narrower the peak, which results in a higher quality factor due to the decreased scattering efficiency^29^. We found it important to show also the peak behavior in case of different grating depths. We extended the Supplementary materials with these informations.

### Experimental demonstration of cut-off sensing

**Figure 13:**
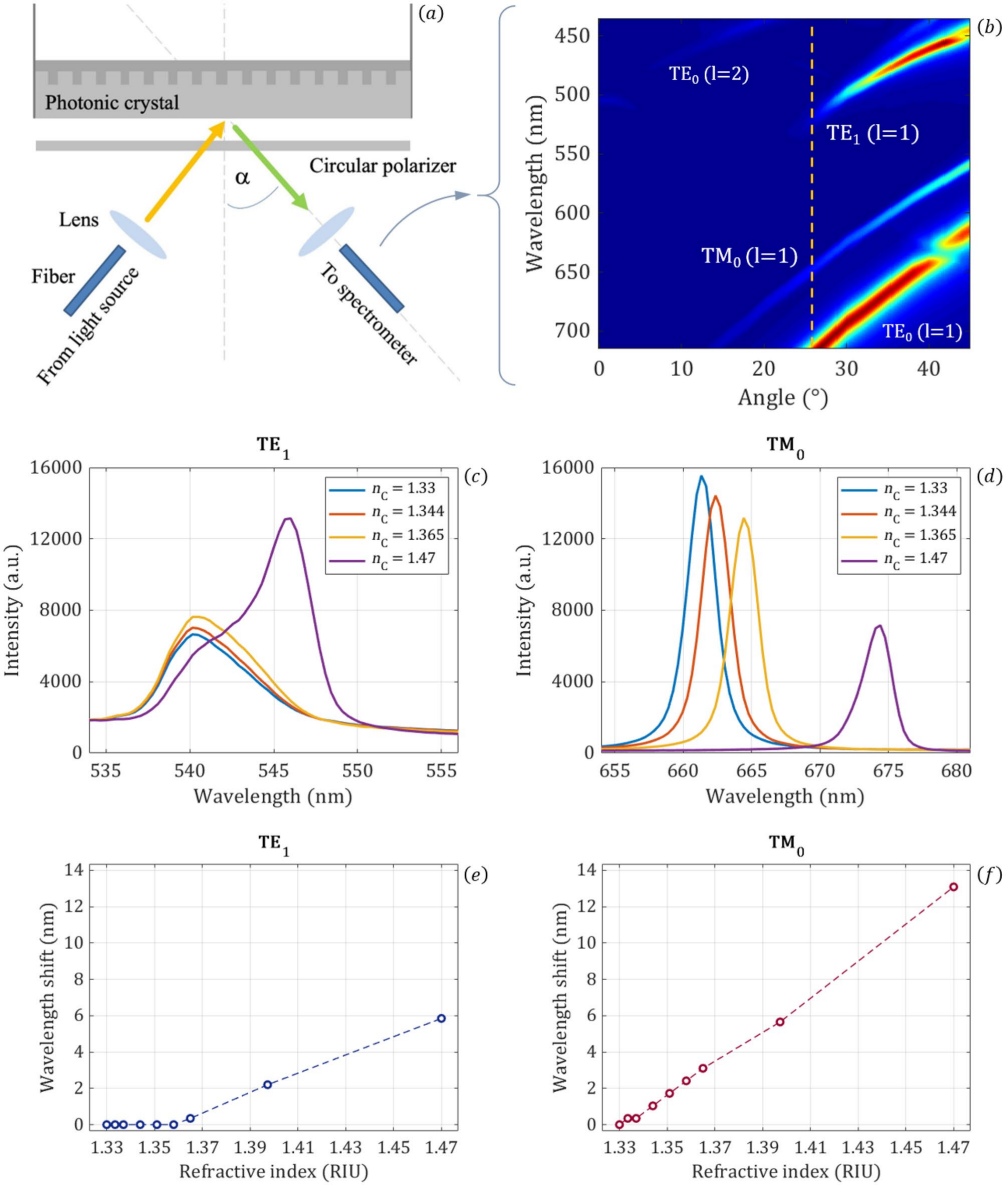
(**a**) Experimental setup for reflection measurements with grating waveguides with an incidence angle of 26°. (**b**) Intensity results as a function of incidence angle and wavelength (normalized to lamp spectrum and interpolated) for n_c_ = 1.33. (**c**) Spectra of TE_1_ mode near cut-off for four different refractive indices and (**d**) spectra of TM_0_ more far-from cut-off. (**e**) Wavelength shift for the near cut-off TE_1_ resonance and (**f**) far from cut-off results for the TM_0_ mode for a refractive index sweep.

To validate the key numerical finding that the intensity decreases near cut-off and the wavelength shift vanishes, we performed experimental reflection measurements on grating waveguides embedded in the bottom of a microplate (Epic 96-well plate by Corning), which enables an easy exchange of the fluid on top of the grating waveguide. The grating waveguides have a periodicity of ~ 500 nm and a high index layer of ~ 120 nm. The experimental setup is shown in Fig. 13a. A halogen lamp is used as excitation light source. It is coupled into a multimode fiber. After exiting the fiber the excitation light is collimated with a collimation lens to a divergence angle of smaller than 1° (checked by beam diameter of ~ 5 cm in projection to a wall in ~ 2 m distance). The incidence angle α of the excitation light is varied. The excitation and reflected light are passed through a circular polarization filter. We used this circular polarization filter to obtain solely the guided-mode resonance and suppress the off-resonance background light^43,44^. The reflected light is coupled with a second lens into a second fiber and guided to a spectrometer. The spectrum is measured from 444 to 796 nm with a resolution of 0.34 nm. Two sets of experiments are conducted. In the first set of experiments the incidence angle is varied from α = 0° to α = 70° in 2° steps for water as the analyte. In the second sets of experiments spectra are measured for a fixed incidence angle of 26° for different analytes. To investigate the wavelength shift with changing refractive index, we used a water-glycerin dilution for tuning the refractive index from 1.33 (pure water) to 1.47 (pure glycerin). In typical biosensors, the refractive index of interest indeed starts at 1.33, as most biological reagents are water based, and goes up to values similar to the refractive index of glycerin.

Figure 13b shows the reflected intensity as a function of incidence angle and wavelength with water as analyte as a heat map. The values are normalized to the lamp spectrum and interpolated. Due to the circular polarizer, both TE and TM resonances are clearly observed and the background is suppressed. The resonances were identified by using a linear polarizer at different angles. It is clearly visible that the TE_1_ wavelength is at cut-off around 26° incidence angle. The mode is not supported at smaller incidence angles and the resonance intensity is reduced upon approaching the cut-off. These results are in line with our intensity results presented in Ref.^26^ and the numerical results in this paper. Compared to the simulation results the experimental results are shifted and the TM_1_ resonance is not observed. This can be explained by the uncertainty in the refractive index properties.

Figure 13c,d show the TE_1_ and TM_0_ spectra for four different analyte refractive indices. These spectra are not normalized to the lamp spectrum and the decreasing lamp intensity for higher wavelengths is clearly visible in the TM_0_ intensity. Note that the change in intensity is highly setup dependent. It would require a more precise calibration to remove the system response. The system response can be used as an advantage to get a higher sensitivity for a given application^33^. Thus, we show the raw data here. The quality factors of the modes at 26° incidence angle are Q_TE1_ ~ 100, Q_TM0_ ~ 300, and Q_TE0_ ~ 40 with the different quality factors also visible in Fig. 13b. The very different behavior of the TE_1_ mode at cut-off and the TM_0_ mode far from cut-off is clearly observed. The TM_0_ mode shifts to the left with decreasing refractive index, while no wavelength shift is observed for the TE_1_ mode near cut-off. Here, only a decrease in the intensity is visible. Figure 13e,f show the wavelength shift obtained by tracking the peak maximum position. The predicted behavior of negligible wavelength shift near cut-off is confirmed for the TE_1_ mode. For the same refractive index sweep the far-from cut-off TM_0_ resonance shows a nearly linear wavelength shift with ~ 90 nm/RIU sensitivity. Compared to the simulation results it needs to be considered that we observe the TE-mode near cut-off, not the TM-mode (which is already in cut-off for the experimental conditions). The quality factor of the TE mode is lower both far-from cut-off and at cut-off than the quality factor of the TM mode. Also, the remaining experimental divergence limits the quality factor. Nevertheless, it is clearly visible in the experimental results that the low-wavelength edge of the TE_1_ resonance is pinned, while the high-wavelength edge shifts further towards the right increasing the quality factor as cut-off is approached. The combined increase in quality factor and reduction of amplitude leads to an overall decrease in the mode intensity as predicted by the simulations.

## Conclusions

We have investigated the near cut-off operation of resonant waveguide gratings for sensing by simulation techniques. Near cut-off and far from cut-off waveguide modes are investigated and their typical characteristics for sensing purposes are compared. The results of the numerical simulations performed by full wave electromagnetic solvers correspond with the simplified analytical calculations.

First, we presented the magnetic field distribution of two resonant peaks, which are suitable for sensing. The electromagnetic field is localized into the waveguide much stronger in case of the reflection peak, which corresponds for the far-from cut-off mode. By contrast, the reflection peak of the near cut-off resonance produces more than three times larger penetration depth into the sensing space. This larger sensing depth allows the sensing of larger biological objects and can monitor the changes of the refractive index further away from the waveguide surface.

Furthermore, parameter sweeps were performed to analyze the guided wavelength and the intensity of the reflection peak for different waveguide thicknesses and cover material parameters. The simulations indicate that new types of sensors can be developed by measuring the intensity near the cut-off point. This type of measurement allows a remarkably high sensitivity for the refraction index variations of the waveguide cover media compared to the traditional wavelength shift measurement. Integrating the guided-mode resonance with light emitting diodes can be one solution to create cost-effective and straightforward sensors based on intensity readout^10^.

We have demonstrated the flexibility of the design, which can be tailored to the needs of different application with different sensitivity and cut-off point by changing the parameters of the waveguide. To support these theoretical results we compared the numerical findings with experimental reflection measurements and found a reasonable match regarding the cut-off behavior.

The proposed sensing mechanism using the near cut-off resonance offers an exceptionally simple design in intensity interrogation measurements. The measurement setup has high sensitivity near to the cut-off point without a need of a wavelength cut-off filter. This compact solution could potentially eliminate vibration noises and it can lead to increased Q-factor. Moreover, such a sensor is an excellent candidate for miniaturization and for low cost production for a wide range of applications.

## Supplementary Information

Supplementary Animation 1a.

Supplementary Animation 1b.

Supplementary Information.

## Data Availability

The dispersion of the waveguide parameter, which is used to support the findings of this study can be found on the webpage of Mikhail Polyanskiy^35^. The other sensor structure parameters are included within the article. Two of the animations of the wave propagations for near cut-off and far from cut-off resonances can also be found in the Supplementary materials.
