# Supplementary figures and images for: Near cut-off wavelength operation of resonant waveguide grating biosensors
Source: Sci Rep. 2021 Jun 22;11:13091. doi: 10.1038/s41598-021-92327-4 (PMC8219702; doi:10.1038/s41598-021-92327-4)

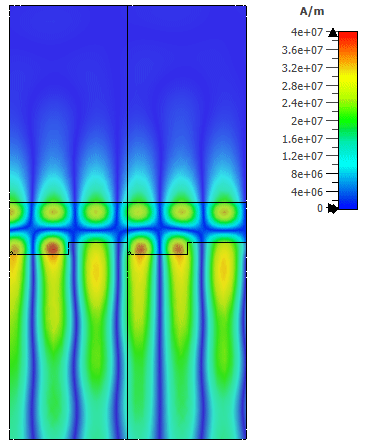

Supplement: Supplementary file 1 — Supplementary Animation 1a. [file 41598_2021_92327_MOESM1_ESM.gif]

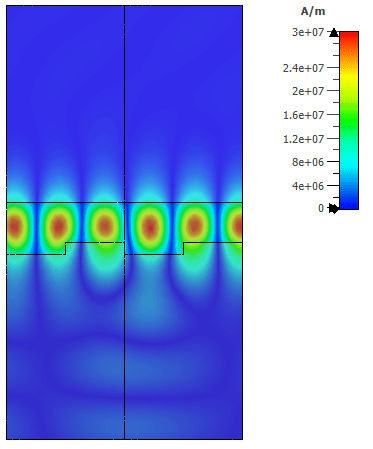

Supplement: Supplementary file 2 — Supplementary Animation 1b. [file 41598_2021_92327_MOESM2_ESM.gif]
