# Supplementary material for: Near cut-off wavelength operation of resonant waveguide grating biosensors
Source: Sci Rep. 2021 Jun 22;11:13091. doi: 10.1038/s41598-021-92327-4 (PMC8219702; doi:10.1038/s41598-021-92327-4)
Supplement: Supplementary file 3 — Supplementary Information. [file 41598_2021_92327_MOESM3_ESM.docx]

***Supplementary Materials***

***Near cut-off wavelength operation of resonant waveguide grating biosensors***

*Balint Kovacs^1,2,3^, Fabio Aldo Kraft^4^ Zsolt Szabo^5^, Yousef Nazirizadeh^6^, Martina Gerken^4^, Robert Horvath^1^*

^1^ *Nanobiosensorics Laboratory, ELKH EK MFA, Budapest, Hungary*

^2^ *Division of Medical Image Computing (MIC), German Cancer Research Center (DKFZ), Heidelberg, Germany*

^3^ *Medical Faculty Heidelberg, Heidelberg University, Heidelberg, Germany*

^4^ *Institute of Electrical Engineering and Information Technology, Kiel University, Kiel, Germany*

^5^ *Faculty of Information Technology and Bionics, Pázmány Péter Catholic University, Budapest, Hungary*

^6^ *Byonoy GmbH, Hamburg, Germany*

**Supplementary materials:**

**1. Supplementary animation 1:**

The animations of the wave propagations for near cut-off and far from cut-off resonances:

- **supplementary** **animation** **1a:** EMF_nearCut-Off.gif: magnetic wave propagation of the TM_1_ guided mode, it is the animation of **fig. 4b**
- **supplementary** **animation** **1b:** EMF_nonCut-Off.gif: magnetic wave propagation of the TM_0_ guided mode, it is the animation of fig. 4e

The magnetic field distribution animations are calculated by CST Studio Suit37.

**2. Effect of grating depth on the near cut-off resonant peak**

As we have already shown in **sec. 3.2** the tuning of $d_{F}$ has a huge effect not only on the location of the cut-off point but also on the sensitivity of the peak intensity. It is already shown that the shallower the grating, the narrower the peak, which results in a higher quality factor due to the decreased scattering efficiency^29^.

Simulations are performed for the grating depths $\sigma=20, 50, 80 nm$, waveguide film thickness $d_{F}=185 nm$ the cover refractive index $n_{C}=1.4$. The reflection peaks and the corresponding magnetic field distributions can be seen in **supplementary** **fig. 1**. The simulations show that the modification of the grating depth leads to the change of the coupling efficiency. For deeper grating depths the resonant peak becomes wider and the coupling is more efficient. The coupling efficiency can be observed as well in the magnetic field distributions of **supplementary** **fig. 1b**, where the localization of the mode power inside the waveguide film is stronger for smaller grating depths.

**
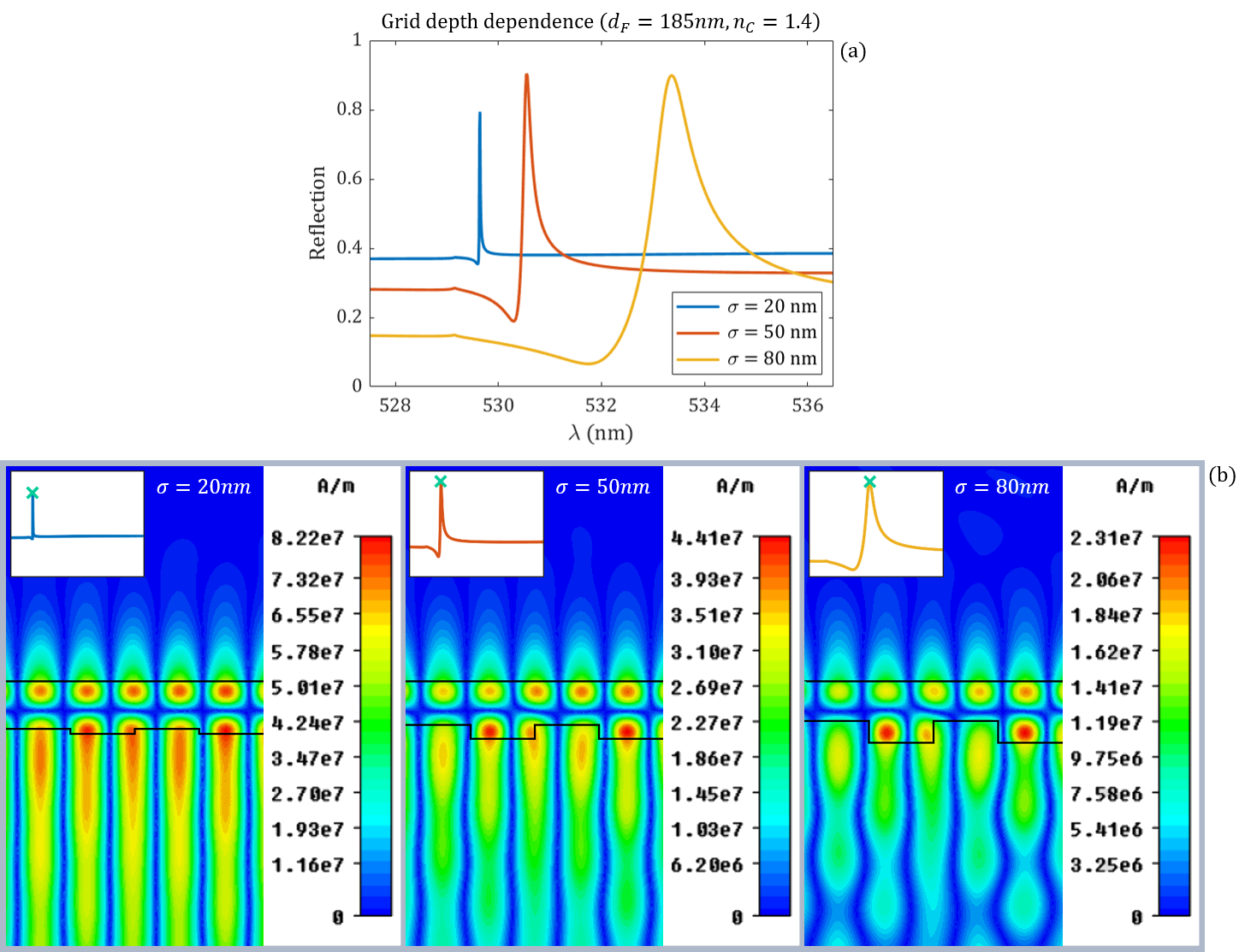
**

**Supplementary** **figure 1:** *The* r*eflection spectra of the near cut-off peak for different grating depth values (a) and the corresponding magnetic field distributions (b). The localization of the mode power inside the waveguide film is weaker for smaller grid depths, which indicates a lower coupling efficiency than those, which correspond to deeper grid sizes. Thus, the quality factor will be higher for shallower gratings. The magnetic field distributions are calculated by CST Studio Suit^37^.*

In **supplementary** **fig. 2** the variation of the peak intensity in function of the cover refractive index $n_{C}$ are shown for several grating depths.


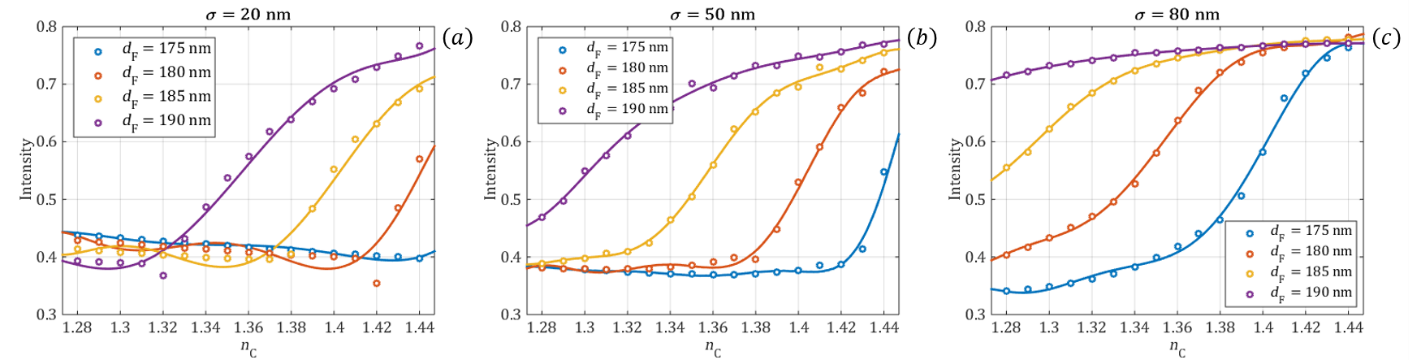


**Supplementary** **figure 2:** *The intensity of the near cut-off peak as function of the refractive index of the cover layer for different waveguide film thicknesses and several grid depth values.*

Other parameters of the waveguide, the material of the waveguide, the grating constant, the refractive index of the substrate is important when the sensor structure is designed and can modify the sensitivity and the cut-off point. However, by changing only two independent parameters the flexibility of the design for different applications is demonstrated.
